# Optimizing Patient Education of Oncology Medications: A Patient Perspective

**DOI:** 10.1007/s13187-018-1406-9

**Published:** 2018-08-03

**Authors:** T Lambourne, LV Minard, H Deal, J Pitman, M Rolle, D Saulnier, J Houlihan

**Affiliations:** 1 Department of Pharmacy, Nova Scotia Health Authority, Halifax Infirmary Site, 1796 Summer St., Halifax, N.S. B3H 3A7 Canada; 2 Department of Pharmacy, Nova Scotia Health Authority, Victoria General Site, 1276 South Park St., Halifax, N.S. B3H 2Y9 Canada; 3 College of Pharmacy, Dalhousie University, 5968 College Street, PO Box 15000, Halifax, N.S. B3H 4R2 Canada

**Keywords:** Oncology, Medication, Chemotherapy, Education, Patient perspective, Focus group

## Abstract

The medication information needs of patients with cancer have been primarily studied using quantitative methods and little qualitative research on this topic exists. The purpose of this study was to explore patients’ perspectives of optimal oncology medication education provided to patients at the Nova Scotia Health Authority (NSHA). Adult (≥ 18 years) outpatients in medical, gynecological and hematology oncology at NSHA were invited to participate in focus groups, which were audio-recorded, transcribed and analyzed thematically. Three focus groups, including 21 outpatients, were conducted. Four major themes were identified: (1) *preparing for what lies ahead* consisted of: readiness to receive information, anxiety over the unknown, setting expectations and patients supporting one another; (2) *bridging the information gaps* was made up of gap in provision of patient education, gap in continuity of patient education, and gap in trustworthy information; (3) *understanding the education needs of the patients* was comprised of sources of information, education timing and setting, prioritizing information needs, and individuality; and (4) *experience within the health care system* encompassed: interactions with health care professionals, willingness to ask questions, patient satisfaction, and financial implications. This study identified previously unknown patient education needs and also supported ideas reported in the literature. This data will guide the strategies that will be used to optimize the delivery of oncology medication education at our facility and other health care institutions.

**Electronic supplementary material:**

The online version of this article (10.1007/s13187-018-1406-9) contains supplementary material, which is available to authorized users.

## Introduction

The provision of oncology medication education is becoming progressively more important due to increasing complexity of cancer treatments, an aging population, and improved prognoses [1, 2]. To optimize patient education, it is important to explore the patient perspective, as this is associated with greater patient satisfaction, improved illness perception, decreased anxiety and depression, and improved quality of life [3–6]. Additionally, there is an association between patient satisfaction with the quality of service provided and survival outcomes in various oncology settings [7–10].

Traditionally, health care professionals decide upon the type, format, and amount of information that is provided to the patient [11]. However, information priorities of health care professionals often do not align with those of the patient [5, 6, 12]. In addition, the information needs of patients undergoing chemotherapy are not static and may change over the course of care [13].

The medication information needs of patients with cancer have been primarily studied using quantitative methods and little qualitative research exists to probe more deeply into the patient perspective. Only one qualitative study conducted in a Dublin cancer center was identified; however, complete study results have not yet been published.

Currently at Nova Scotia Health Authority (NSHA), patients typically receive education from their oncologist at diagnosis, from nurses during chemotherapy treatment and a small number of patients may receive education from a pharmacist. Very few patients are educated by a pharmacist due to a number of barriers, which include limited oncology pharmacist staffing, and the high volume of patients and chemotherapy orders received. The current Canadian Standards of Practice for Oncology Pharmacy recommend that oncology pharmacists should be providing complete medication education to patients [14]. It is currently unknown what oncology medication education patients at NSHA wish to receive. Therefore, the objective of this study was to explore patients’ perspectives of optimal oncology medication education provided to patients at NSHA.

## Methods

### Study Design

A qualitative study design, using focus groups, was selected to address the research objective. Focus groups perform well when the objective is to examine perceptions around issues, programs, or services and can aid in developing new programs or services [15].

### Participants

Medical oncology, gynecology oncology, and hematology oncology adult (≥ 18 years or older) outpatients at NSHA who received ≥ 75% of their prescribed chemotherapy protocol, completed at least three cycles of chemotherapy, were currently receiving second or greater line of chemotherapy, or had completed treatment up to 3 months prior to screening were invited to participate via a patient information sheet provided by nurses in the chemotherapy treatment rooms. Patients from across the province who received intravenous chemotherapy at the Victoria General Hospital in Halifax, Nova Scotia, Canada were included in the study. Patients may also have received oral chemotherapy. Chemotherapy was broadly defined to include treatment with immunotherapy, hormone therapy, targeted, or cytotoxic agents.

### Focus Group Moderation

Focus groups were moderated by the principal investigator and assistant moderator. All participants received an information package prior to participating, and completed a demographic data form, which included their age, sex, education level, marital status, and living situation. Both the principal investigator and assistant moderator signed a pledge of confidentiality.

### Data Collection and Analysis

The focus group questioning route (Online Resource 1) consisted of short, open-ended questions that were pilot-tested by research team members, non-researchers, and ten patients who met eligibility criteria. Focus groups were audio-recorded and transcribed and data analysis was assisted by NVivo 11 software [16]. Field notes were completed by the principal investigator and assistant moderator to describe the physical setting, nonverbal communication, key words or phrases expressed by participants, and the sequence of speakers [17]. After each focus group, the principal investigator and assistant moderator had a debriefing session, which is a 15–20 minute audio-recorded oral review of the most important themes or ideas discussed [15, 18]. Data was analyzed thematically using the constant comparative method to continuously compare responses of participants in each group [15]. Through iterative cycles, participants’ responses were coded, related codes were grouped together to form categories, and themes were developed, which represented related categories [17, 19]. Thematic analysis was conducted by the principal investigator and reviewed by two co-investigators.

### Rigor and Trustworthiness

Several steps were taken to ensure trustworthiness of the data. A reflexive journal was used to keep a record of thoughts and codes and to recognize any assumptions and biases that could affect the data analysis process [17, 19, 20]. An audit trail was created by the principal investigator describing the decisions made in the development of codes, categories, and themes. Participant quotes are presented to show the relationship between the original data and the categories and themes that were developed [19].

## Results

Three focus groups, including a total of 21 oncology outpatients, were conducted in January 2017 at the Victoria General Hospital. There were six to eight participants in each focus group. Participants’ ages ranged from 51 to 82 years (mean 65 years). Education levels of participants included college or university, high school, and junior high school. Participants’ marital statuses included married, divorced, common-law, single, and widowed. Additional demographic and clinical characteristics are presented in Table 1.

**Table 1:** Demographic and clinical characteristics of focus group participants including sex, living situation, diagnosis, chemotherapy route of administration, and mean number of chemotherapy cycles

	Focus groups (*n* = 21) *n* (%)
Sex	
Male	9 (43%)
Female	12 (57%)
Living situation	
Living with spouse/family/friend/other	15 (71%)
Living alone	6 (29%)
Diagnosis^a^	
Solid tumor	15 (71%)
Hematological tumor	6 (29%)
Oral chemotherapy	9 (43%)
Intravenous chemotherapy	21 (100%)
Mean number of chemotherapy cycles (range)	18 (4–83)

^a^Diagnoses included multiple myeloma, lymphoma, breast cancer, prostate cancer, lung cancer, renal cell cancer, colon cancer, ovarian cancer, and melanoma

Four major themes, 15 categories, and 67 codes were identified. A comparison of focus group codes is presented in Table 2. The four major themes included *preparing for what lies ahead*, *bridging the information gaps*, *understanding the education needs of the patients*, and *experience within the health care system.*

**Table 2:** Number of participants, number of new codes identified, and total number of codes identified within each focus group

	Focus group			
	A	B	C	Total
Number of participants	6	8	7	21
Number of new codes	61	5	1	67
Total number of codes	61/67 (91%)	62/67 (93%)	62/67 (93%)	67/67 (100%)

### Preparing for What Lies Ahead

#### Readiness to Receive Information

Oncology patients described feeling overwhelmed when discussing their experiences receiving information upon diagnosis. Not feeling ready to listen to or absorb information was common because patients needed time to come to terms with their diagnosis. Patients felt that they were given too much information at one time, that everything happened very quickly, and that they were unable to absorb the information provided: “the doctor could have even told me but in the initial meeting with her, like, I went blank when she started telling me all this stuff, it’s just like-overwhelming-so much information” (participant B1). Patients emphasized the importance of having family members/friends present during appointments. For example, participant A3 commented: “I like to bring a friend [to appointments], because it can be very overwhelming and you don’t know the questions to ask.”

#### Anxiety over the Unknown

Not knowing what to expect was a significant source of fear, worry, and vulnerability for patients. Worrying about whether a particular sign or symptom was being experienced caused anxiety among patients: “I think you read [the side effects] and you think oh my god…do I have that? And then it stays with you – ‘cause your mind is playing games on you right ‘cause-you’re just sometimes not in a good place-you’re sitting there thinking, am I getting this?” (participant C4).

#### Setting Expectations

Patients identified information they would have liked to know up front to set expectations. Knowing that chemotherapy is individualized was one concept many patients identified: “I noticed some people stay a long time to have chemotherapy whereas mine was over a short time so I asked the nurse why was this, was the drug not working so well? She said probably not, it’s probably a different treatment than yours” (participant B5). Other information patients identified that they would have liked to know up front included prevalence of side effects, expected effect of treatment on the disease, and the potential duration of treatment.

#### Patients Supporting One Another

Oncology patients felt that sharing stories with other patients helped to provide reassurance as well as a sense of community or family. For example, participant A2 commented: “I found speaking with other people and sharing stories was very helpful too and, I mean, you know, you’re in kind of a vulnerable position there so I think everybody’s pretty open at that point because we all know what we’re going through” (participant A2).

### Bridging the Information Gaps

#### Gap in Provision of Patient Education

Patients identified that health care professionals are busy and, in particular, emphasized a lack of contact with the pharmacist: “I only saw a pharmacist here in the hospital once for maybe like 30 seconds…so that certainly wasn’t the person who was educating me on my chemotherapy medicines” (participant B3).

Patients indicated there was a lack of education on chemotherapy drugs, which included drug interactions, how the drug works, the effect of chemotherapy on blood work, chemotherapy precautions, and a lack of guidance on internet resources. Patients also identified a lack of education on side effects and information on complementary cancer therapies, vitamins, and herbs. Participant B4 described a situation where she experienced a lack of education on side effects:

“They don’t tell you about any side effects, I’m not talking about all side effects, I’m talking about the most common maybe, what to look for? I’ve had occasions where we didn’t go to the hospital right away and I had a pulmonary embolism and I laid down for a day and a half at home and when I decided to go to the hospital the doctor was like: why didn’t you come and did you know that this is dangerous?”

#### Gap in Continuity of Patient Education

Oncology patients identified that reiteration of information was crucial because patients often had difficulty remembering what was said.

I think the more information you get and the more frequently it’s given and throughout the whole process like-at various stages throughout it was reiterated that this is what you’re getting and this is the side effect (participant A2).

Reiteration! That’s the word right there (participant A3).

Patients identified they would like education on their chemotherapy medications to be continuous, with the opportunity to get more information with each treatment. Patients also emphasized the importance of receiving ongoing follow-up.

#### Gap in Trustworthy Information

Patients exhibited a lack of trust with internet sites or resources and wanted to receive more trustworthy resources from their health care professionals. For example, participant A2 commented: “you don’t want to be reading some dumb blog when cancerresearch.co.uk is where you really need to be.”

Patients wanted to receive open and transparent information on their chemotherapy medications. Some patients felt that they did not always have complete information and that “there was this-this secrecy surrounding it like they would only let you know what you need to know” (participant B3).

### Understanding the Education Needs of the Patients

#### Sources of Information

Patients identified the importance of having information they could refer back to. In particular, patients liked having written information to take home with them, but also mentioned medication calendars, keeping their own notation in a patient logbook and having the ability to record conversations.

When asked about which ways patients preferred to receive information on their chemotherapy drugs, most indicated they would like a combination (e.g., hearing the information, written information, video, medication calendar). Patients indicated that they would like to receive more quantitative information, such as prevalence of side effects and percent cure: “Nobody ever tells you the percentage of the good results from the drug… I think somebody should” (participant C2). When the groups were asked what their opinions were on scheduling a separate appointment to meet with the pharmacist, all but two patients indicated they would be interested in doing so.

#### Education Timing and Setting

Although there was no clear consensus on the exact timing of education, most patients identified that they would like to receive *some* information up front (e.g., before or immediately after starting treatment): “then when you go in for the first time, you go in to have your treatment, you have a little bit, and you’ve had time to look at what you are going to do and then you know what type of questions to ask” (participant B4). Patients indicated that they would like to receive follow-up shortly after their chemotherapy treatments (e.g., a few days to a week after treatment) to allow time to process information and bring up any issues encountered with their treatment.

In terms of education setting, most patients preferred to have one-on-one education sessions with a health care professional. However, a few patients also commented that a group session would be helpful, particularly if all patients in the group were getting the same treatment. In terms of location, some patients preferred in-person meetings while others preferred a telephone call. Patients did note that the timing and setting of education sessions “depends on their health and ability to get to the hospital” (participant B3).

#### Prioritizing Information Needs

One question from the focus group questioning route (Online Resource 1) asked participants to list the top five education items they felt were most important to know about their chemotherapy drugs. Participants received a list of potential education items but were strongly encouraged to come up with their own. In addition to this question, participants also brought up education items independently throughout the focus groups. The top nine education items are presented in Table 3.

**Table 3:** Top nine education items identified by focus group participants as being most important to know about their chemotherapy medications

Education item	Information on side effects^a^
	How and when to take the medication
	How the drug works
	Drug interactions
	Alarm symptoms and who to contact
	Effects of treatment on lifestyle
	Storage, handling, and disposal of the medication
	Education around anti-nausea drugs
	Information on insurance, drug coverage, and cost of the medication

^a^Identified by participants as the most important education item, and includes serious versus common side effects, short- versus long-term side effects, and the prevention and management of side effects

#### Individuality

The importance of individualizing patient education was emphasized throughout the focus groups. Patients expressed that “education is personal” (participant B4) because “every person is totally different” (participant C4) and has “different learning styles” (participant B3).

### Experience Within the Health Care System

#### Interactions with Health Care Professionals

There was discussion around which health care professional provides what type of information and patients commented that the information provided often overlaps. Patients expressed that communication between health care professionals and having “that team effect” (participant C7) was important. Patients felt that new vocabulary, particularly the drug names, were often confusing and that knowing both the brand and generic name of drugs would help lessen confusion.

Patients discussed the importance of health care professionals having “that personal touch and establishing a relationship” (participant C7) with the patient. Patients also expressed the importance of being an active participant in the treatment decision. One patient described their experience where this was not the case: “I was never given the option. I was told this is what you’re getting and this is what’s going to happen. Take it or leave it” (participant B6).

#### Willingness to Ask Questions

When patients were asked whether they felt comfortable asking questions and if they had adequate opportunity to do so, most answered yes. However, patients expressed they would feel more comfortable asking questions in a private area. It was also emphasized that patients would be more willing to ask questions if health care professionals, in particular pharmacists, were present on the floor. For example, Participant C2 commented: “if pharmacy could somehow get on the floor the same as the nurses are and maybe visit people and if somebody does have a question they’re more willing to ask if you’re right there with them.”

#### Patient Satisfaction

Patients appreciated the care and information they received from their nurses, oncologists, pharmacists, and family doctors. For example, participant C3 commented: “I just would give up I think, if it wasn’t for the nurse. We don’t realize how important the nurses are, until you have to go through something like this.” Patients were satisfied that improvements to oncology patient care were being made. However, frustration was expressed when patients discussed the long wait times to receive their chemotherapy.

#### Financial Implications

Patients identified cost of medications as a major concern. Although patients acknowledged that “a lot of money is invested in us” (participant A2), they also expressed frustration when discussing that “if the drug is given intravenously here then it’s covered, but if you’re taking a pill it’s not covered” (participant A3). Patients emphasized the need for more pharmacy staff. Participant B7 identified that “the government is going to have to finance or pay for more pharmacists.”

## Discussion

### Preparing for What Lies Ahead

As previously described in the literature, patients may not adequately process and retain the information they receive until they are ready to accept their cancer diagnosis [13]. Another finding supported by the literature, which was voiced by participants during focus groups, was the importance of having family or friends present at appointments [21]. Explaining that chemotherapy regimens are individualized for each patient was a patient education need that was identified, which could help lessen anxiety and confusion among patients. An unexpected finding was that “hearing the experiences of other patients” and “knowing what others were going through” was described by patients as one of the most important parts of the focus group discussion. Access to patient support groups should be an integral component of education programs.

### Bridging the Information Gaps

Adequately addressing the information needs of all patients is challenging, given the rising demands on health care professionals and the limited time allotted to outpatient appointments [22]. This was recognized by patients and is likely a contributing factor to the gap in provision of patient education that was identified. A gap in continuity of patient education was described, with the finding that patients would like to receive education sessions continuously throughout treatment. Patients stressed the importance of repeating information to improve retention, as well as the desire for trustworthy internet resources, which has been recommended previously [2].

### Understanding the Education Needs of the Patients

Patients identified they would like to receive a combination of different information modalities but emphasized the importance of written information they could refer back to, an idea which has also been supported in the literature [2, 13]. Our results demonstrate that patients would like to receive more quantitative information including prevalence of side effects and expected effect of treatment on the disease. The identification of the top nine education items prioritized by patients will aid in the development of a new education model. However, it was emphasized in our study and previous research that education should be tailored to meet the needs of the individual patient [11, 21].

### Experience Within the Health Care System

Moving towards a patient-centered instead of a disease-centered approach is important, as patients want to be active participants in the treatment decision [23]. It has been recommended that education take place in a private setting, which was also identified by patients in our study [21]. Patients discussed system issues, which included intravenous versus oral chemotherapy drug coverage, wait times for chemotherapy and the need for more pharmacy staff. Decision makers must address these issues in order to improve cancer care.

### Strengths and Limitations

Using a qualitative study design enabled the collection of rich, detailed information from patients. The purposive sample consisted of patients in later phases of treatment or who have completed treatment because these patients have more experience receiving oncology medication education. Patients with a wide variety of cancer diagnoses were included in this study, which helps increase transferability of the findings. It was estimated that three to six focus groups consisting of four to eight participants per group would be needed to reach theoretical data saturation [15, 24]. The idea that theoretical data saturation was achieved was supported by the identification of only one new code after the last focus group.

Although patients with a wide variety of diagnoses and stages of disease participated in the study, it is possible that the sickest patients may not have been represented. In addition, it was noted by nursing staff that many interested patients were unable to participate due to travel constraints.

### Relevance to Practice and Future Directions

Results of this research will guide the strategies that will be used to improve the delivery of oncology medication education to patients at our facility and other health care institutions. Further research is required to determine if the results of this research apply to a broader population, as well as to delineate the role of each health care professional in providing oncology patient education.

## Electronic Supplementary Material

ESM 1 (PDF 81 kb)
