# Supplementary material for: Optimizing Patient Education of Oncology Medications: A Patient Perspective
Source: J Cancer Educ. 2018 Aug 3;34(5):1024–30. doi: 10.1007/s13187-018-1406-9 (PMC6785581; doi:10.1007/s13187-018-1406-9)
Supplement: Supplementary file 1 — (PDF 81 kb) [file 13187_2018_1406_MOESM1_ESM.pdf]

Optimizing Patient Education of Oncology Medications: A Patient Perspective

Journal of Cancer Education

Lambourne T[1], Minard LV, Deal H, Pitman J, Rolle M, Saulnier D, Houlihan J

[1]Corresponding author: Tessa Lambourne; Department of Pharmacy, Nova Scotia Health Authority, Halifax

Infirmary Site; E-mail: tessa.lambourne@nshealth.ca

Online Resource 1: Focus Group Questioning Route

Question 1 (Opening-5 minutes)

-Let's get started. We will begin by asking everyone around the table to introduce themselves. If you are not comfortable sharing your first name please feel free to make one up if you like!

-Tell us your first name, and how long you have received chemotherapy.

Question 2 (Introduction- 15 minutes)

-I know that you have all received lots of different types of information throughout your treatment. I would just like to emphasize again that we will be focusing on education you have received on your chemo drugs only.

-Tell us about your experience receiving education on your chemo drugs.

Prompt

-What source of information was used to provide education on your chemo drugs? Examples include: hospital staff, written information, watching a video, other patients, looking it up on your own on the internet.

-What type of information did you receive about your chemo drugs? Some examples include: how the medication works, how and when the medication is given, side effects and their prevention and management and drug interactions.

Question 3 (Key- 5 minutes)

-Think back to a time when you received education on your chemo drugs.

-Is there a time during your course of treatment that would have been better to receive education on your chemo drugs?

Prompt

-Examples of times you could have received education include: when you were first diagnosed, before your first chemotherapy treatment, at your first chemotherapy treatment, at other chemotherapy treatments, when you experienced side effects.

-How often would you have liked to receive education on your chemo drugs?

Question 4 (Key- 5 minutes)

-There are many different ways to receive information about your chemo drugs. A few ways to receive information include: one on one education, education in a group with other patients, written information, watching a video, hearing a tape or learning about medications on a computer.

-What ways do you/would you prefer to receive information about your chemo drugs?

Question 5 (Key-5 minutes)

-What is your opinion on scheduling a separate appointment to meet with the hospital pharmacist to receive education on your chemo drugs?

Prompt

-How would you like to meet with the hospital pharmacist? For example: by an in-person appointment or by a telephone appointment?

Question 6 (Key-5 minutes)

-How would you feel about receiving follow-up from a hospital pharmacist after you have received education on your chemo drugs? For example: at your next treatment visits or by telephone.

Prompt

-When would you have liked to receive follow-up? For example: by telephone days or weeks after your education session, at your next treatment visits.

Question 7 (Key- 20 minutes)

-What type of information is the most important to know about your chemo drugs?

-Here is a list of possibilities. If there is something not mentioned on this list that you feel is important please write it down.

Type of information to receive about chemo drugs

• How the medication works<br>• Expected effect of treatment on disease<br>• How and when to take the medication<br>• Side Effects<br>• Prevention of side effects<br>• How to manage side effects<br>• Who to contact if experience serious side effect<br>• Information on complementary cancer therapies/vitamins/herbals<br>• Long-term effects of treatment on daily functioning	• Drug Interactions<br>• What to do if you miss a dose<br>• Storage, handling and disposal of medication<br>• Strategies to remember to take your medications<br>• Information on insurance, drug coverage and cost of medication<br>• Effects of treatment on social/family life<br>• Effects of treatment on physical appearance<br>• Effects of treatment on sexual function/activity<br>• Effects of treatment on ability to go to work

-List the top 5 things you think are most important to know about your chemo drugs.

-Let's discuss what everyone thinks is the most important information to know. Explain why you chose the items included in your top 5.

-Where do we agree?

-Are there differences of opinion in our group?

Question 8 (Key- 15 minutes)

-Think back to your experience receiving education on your chemo drugs.

-How could education on your chemo drugs have been improved?

Prompt

-What aspects of the education you have received on your chemo drugs have been difficult to understand?

-Do you feel that you had adequate opportunity to ask questions about your chemo drugs?

-Do you feel comfortable asking questions?

-If no, what would help make you feel more comfortable asking questions?

Question 9 (Ending-5 minutes)

- What do you think the most important parts of this discussion have been?

Question 10 (Ending-5 minutes)

-The purpose of this study was to find out what the best timing and content of education on your chemo drugs is from your perspectives.

-Have we missed talking about anything?
